# Sensory-level electrical stimulation in children with cerebral palsy: a scoping review of current applications and outcomes

**DOI:** 10.3389/fped.2025.1644547

**Published:** 2025-11-24

**Authors:** Céline de Araujo, Alisa Gschaidmeier, Miriam von Gunten, Sebastian Grunt

**Affiliations:** 1 Division of Neuropaediatrics, Development and Rehabilitation, Department of Paediatrics, Inselspital Bern, University Hospital, University of Bern, Bern, Switzerland; 2 Graduate School for Health Sciences, University of Bern, Bern, Switzerland; 3 Department of Physiotherapy, Inselspital Bern, University Hospital, Bern, Switzerland

**Keywords:** cerebral palsy, electrical stimulation, sensory afferent electrical stimulation, spasticity, neuromodulation

## Abstract

**Introduction:**

This scoping review aims to map the existing literature on sensory-level electrical stimulation (ES) interventions for children with cerebral palsy (CP).

**Methods:**

MEDLINE, Embase and PEDro were searched. Among 504 screened articles, 18 studies were included. ES forms utilized included transcutaneous electrical nerve stimulation (TENS), therapeutic electrical stimulation (TheES), threshold electrical stimulation (ThrES), Mesh-Glove Stimulation, and the Mollii suit. The reviewed ES modalities were used in upper extremities, lower extremities, and whole-body treatments.

**Results:**

Some significant improvements were noted in motor control, spasticity, strength, and functional abilities.

**Discussion:**

While the findings suggest that sensory-level ES holds promise for enhancing motor function in children with CP with significant improvements shown in the relevant outcome measures in fourteen of eighteen papers, the existing literature is characterized by significant variability in stimulation parameters, study design, sample sizes, patient characteristics and outcome measures hindering the ability to generalize findings. Further research with larger, more homogeneous samples and standardized protocols are essential to validate these interventions and establish effective treatment guidelines.

## 1 Introduction

Cerebral palsy (CP) is an umbrella term describing a diverse group of movement disorders resulting from pre-, peri- or postnatal, non-progressive lesions or malformations in the developing brain and affecting approximately 2 per 1,000 live births (1, 2). In addition to motor impairments, children with CP often experience a range of comorbidities, including cognitive and sensory deficits as well as epilepsy (1, 3). These limitations frequently impact daily activities and restrict participation in various social contexts. As currently no cure exists for CP, treatment is primarily symptomatic, focusing on enhancing quality of life and fostering greater social integration. Given the heterogeneity of CP, therapeutic approaches are highly individualized to accommodate the wide spectrum of symptoms. Numerous interventions have been rigorously studied in children with CP, showing benefits that extend beyond motor improvement with positive effects on activity levels and participation in activities of everyday life. Physical and occupational therapies are typically foundational, with a strong emphasis on stretching paretic limbs as well as enhancing mobility, supporting functional abilities, and mitigating spasticity in order to provide the highest possible quality of life (1, 4–6).

In addition to traditional therapeutic methods mentioned above, electrical stimulation (ES) is an emerging area of interest in CP treatment (1). ES encompasses a range of techniques applied across diverse patient populations, including pain management and neurorehabilitation for movement support in those with impaired mobility (7). Although originally developed for individuals with spinal cord injuries, these principles of stimulation are increasingly being applied to children with CP, though usage remains limited due in part to lack of experience in this area (8). Recent studies have explored the benefits of Functional Electrical Stimulation (FES), which generates muscle contractions to assist with specific, often impaired, functions. Wright et al. (9) demonstrated significant improvements in hand function in children with CP following a six-week course of FES. An alternative approach, Neuromuscular Electrical Stimulation (NMES)—which elicits contractions without a functional goal—has also shown therapeutic promise. Ozer et al. (10) reported that children receiving NMES with dynamic bracing experienced notable improvements in strength, posture and control over their upper extremities compared to a control group without this combination.

Within the scope of ES, sensory afferent electrical stimulation (SAES) represents a specific technique whereby peripheral electrical stimuli are used to activate action potentials in afferent nerve fascicles without inducing muscle contraction. This leads to increased input to the sensory and motor cortices, thereby creating neuromodulation at a synaptic level within the motor cortex (7). This sensory-level stimulation is an umbrella term and is administered under various names and parameters. These include methods such as transcutaneous electrical nerve stimulation (TENS), therapeutic electrical stimulation (TheES), threshold electrical stimulation (ThrES), and Mesh-Glove Stimulation, as well as incorporated into therapies such as the Mollii Suit. TENS, a versatile modality, can offer benefits such as pain relief, enhanced muscle function, and reduced spasticity and can be categorized into sensory and motor-level applications based on whether or not it induces muscle contractions (11, 12). Mesh-Glove Stimulation applies sensory-level stimulation over the entire hand, enhancing limb awareness, movement, and reducing hemineglect as well as hypertonia (13). ThrES, a low-intensity form of stimulation found at the sensory threshold is to be used over longer therapeutic intervals, for example overnight, and is theorized to prevent disuse atrophy and support muscle growth through improved local blood flow and trophic hormone release during sleep (14, 15). TheES is described as a low amplitude, sensory stimulation that does not cause muscle contraction and which is said to prevent disuse muscle atrophy through increased muscle bloodflow (14, 16). The Mollii Suit, a wearable device containing 58 electrodes, is designed to reduce spasticity by stimulating antagonist muscles and allowing customizable low-level stimulation across the body (17). This diverse collection of terminology with in part overlapping aspects as well as stimulation parameters with lacking standardized definitions provides a challenge in synthesizing results and guaranteeing a comprehensive inclusion of the relevant studies, which will be discussed in more detail later in the paper.

Various forms of SAES have been studied in children with CP for different indications and through a variety of approaches. As these approaches differ significantly in their treatment protocols and associated outcomes, the resulting body of literature is complex and fragmented. To date, there has been no comprehensive summary focusing on the applications and effects of SAES in children with CP.

The primary objective of this scoping review is to systematically assess and map the available literature on sensory-level ES in the treatment of children with CP. Due to the diversity of stimulation methods and parameters, such as frequency, intensity, and pulse duration, this review will examine the range of approaches currently in use. Additionally, it will assess how SAES is administered, including the duration and frequency of treatments as well as targeted anatomical areas. Key aspects to be looked at include outcome measures, CP subtypes, and whether SAES has shown efficacy in symptom improvement. Given the limited and heterogeneous nature of research in this area, a scoping review will serve to organize existing knowledge, summerize the potential benefits of SAES, and determine whether further studies are warranted to support the integration of SAES into clinical practice for CP management.

## 2 Methods

### 2.1 Study design

This paper was classified as a scoping review and was guided by the Preferred Reporting Items for Systematic reviews and Meta-Analyses extension for Scoping Reviews (PRISMA- ScR) (18). In addition, it was conducted using the methodological framework provided by the Joanna Briggs Institute (19). A scoping review was deemed appropriate for this paper due to its effectiveness in mapping the breadth of available research in response to broader questions (20). The protocol for this review is publicly accessible on Open Science Framework (21).

### 2.2 Eligibility criteria

Studies were eligible for review if they met the following inclusion criteria: Research to be included must be 1) full text journal articles examining the treatment of 2) children under the age of 18 with 3) a diagnosis of cerebral palsy, who have 4) undergone sensory level ES applied transcutaneously to skeletal muscles not resulting in muscle contraction. Articles must be written in English or German due the linguistic capabilities of the reviewers in question. All studies published before the date of the final search were included. The start date for the search was left open, and all articles that fulfilled the inclusion criteria up until the search date were incorporated.

### 2.3 Information sources

Research was collected from three online databases: Ovid via Medline, Embase via Elsevier and PEDro: The Physiotherapist Evidence Database, as well as through citation searching. Final searches were completed on the 4th of January 2024; the results were then imported into Mendeley Reference Manager, where duplicates were removed, leaving a total of 338 abstracts.

### 2.4 Search strategy

We developed the search strategy in consultation with a medical information specialist. Preliminary searches contributed to the selection of search terms. These search terms were adapted to each individual database (Supplementary Table A1). Text words and subject headings were combined using the Boolean operators AND and OR. No limits were applied to the search to minimize the exclusion of relevant data.

### 2.5 Selection process

After importing the results of the searches into Mendeley Reference Manager, deduplication was completed. Two reviewers (CdA and AG) then screened the materials independently from one another using the inclusion and exclusion criteria listed above, first according to title and abstract, and at a later stage after reading the full text. Any inconsistencies that occurred were resolved through discussion (CdA and AG). In the case of persistent differences of opinion, a third reviewer (SG) was consulted in order to reach a consensus.

To evaluate the breadth of research available, this scoping review considers both experimental and quasi-experimental study designs including randomized controlled trials (RCT), non-randomized controlled trials, before and after studies and interrupted time-series studies. In addition, analytical observational studies including prospective and retrospective cohort studies, case-control studies and analytical cross-sectional studies have been reviewed for inclusion. This review has also evaluated descriptive observational study designs including case series, individual case reports and descriptive cross-sectional studies for inclusion.

**Figure 1:**
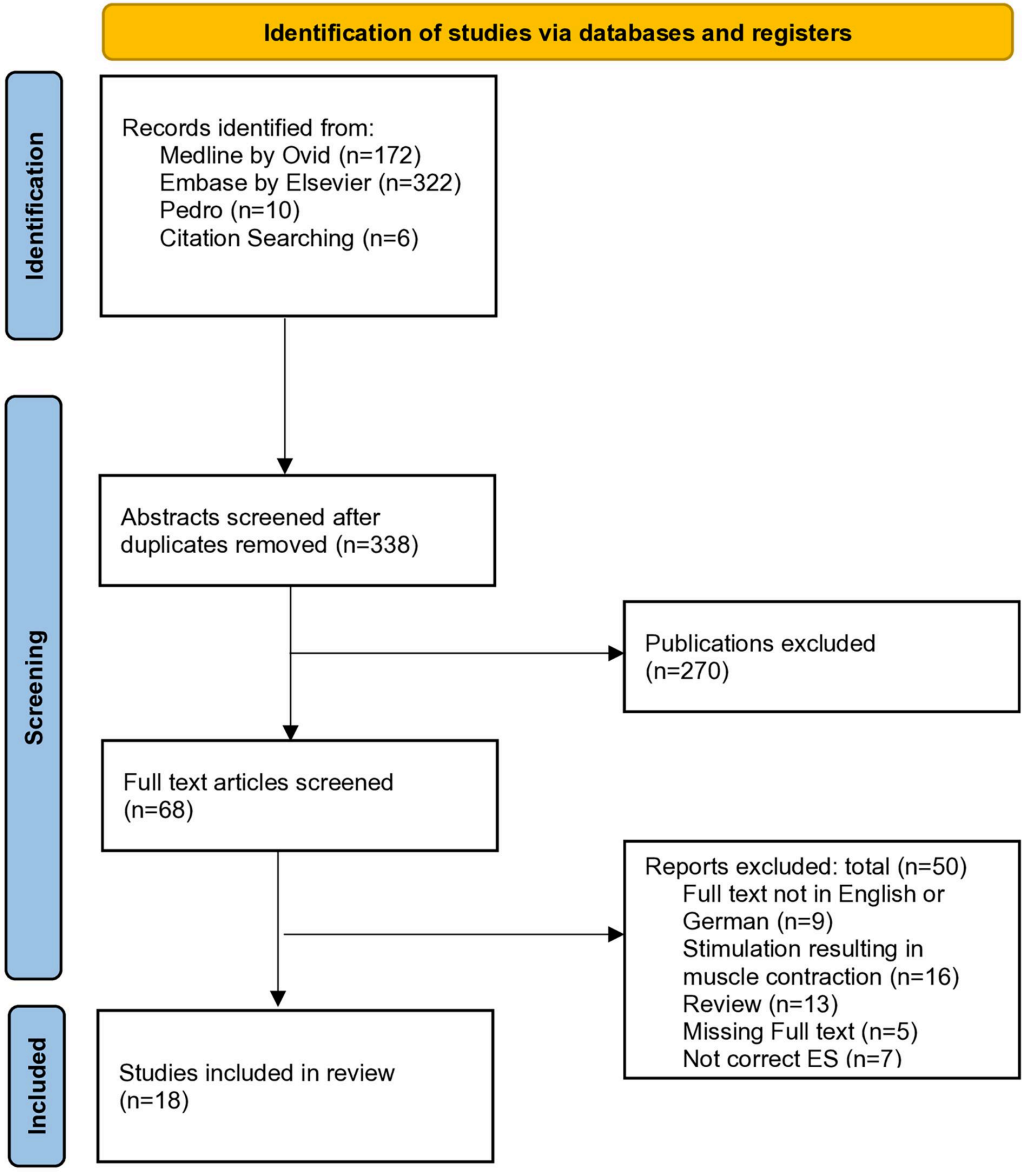
PRISMA flow diagram of the study selection. Source: Page MJ, et al. BMJ 2021; 372:n71. doi: 10.1136/bjm.n71.

Reviews (systematic reviews, literature reviews, scoping reviews and similar) as well as meta-analyses have been included; however, to avoid evaluating the same patient populations multiple times, they were excluded after hand searching the references and extracting relevant articles. The PRISMA Flowchart for the selection process is shown in Figure 1.

### 2.6 Data charting process

The primary data extraction was conducted by the first reviewer (CdA), while the second reviewer (AG) contributed to the extraction of three studies to help minimize bias and reduce the risk of errors in data transfer. The full data extraction table is included in the online supplement, with the condensed version shown in Table 1.

**Table 1:** Characteristics of studies included in this review.

Author (year published)	Study design and main objective	Participants: diagnosis (mean age)	Stimulation location	Intervention with parameters: pulse duration, frequency, intensity	Duration of treatment	Outcome parameters	Significant results
Alabdulwahab et al. (2010)	Prospective Randomized Controlled Clinical Trial Aim: To study the short and relatively long-term effects of conventional TENS on spasticity of hip adductors and gait parameters	SG: 27 spastic diplegic CP children (10.22 years old) CG: 15 TD (9.2 years old)	LL: Adductor longus muscle	TENS: 250 µs, 100 Hz, enough to cause tingling	Short Term: 1× Long Term: 15 min 3×/day for 1 week	Clinical Assessment: Step width, length and speed Spasticity: MAS Muscle Function: Knee position and Gait Assessment	MAS, Knee Position, Step length and speed after short term treatment significant
Alhusaini et al. (2019)	Prospective Randomized Controlled Clinical Trial Aim: To determine whether TENS combined with therapeutic exercises helps to improve hand function by reducing spasticity	SG: 15 hemiplegic CP children (8.5 years old) CG: 14 hemiplegic CP children (8.4 years old)	UL: wrist and finger extensors	TENS: 250 µs, 100 Hz, 50 mA (to cause tickling sensation)	30 min 3×/week for 8 weeks	Strength: Handgrip Unimanual handfunction: Jebsen Taylor Hand Function Test Parents Assessment: ABILHAND Kids	Handgrip and JTHFT
Azzam et al. (2012)	Randomized Controlled Clinical Trial Aim: To show the effect of mesh glove sensory stimulation on spasticity control	SG: 15 hemiplegic CP children (5.33 year old) CG: 15 hemiplegic CP children (5.67 year old)	UL: whole hand + dorsal/ventral aspects forearm	Mesh Glove: 300 µs, 20–40 Hz, 2–10 mA (to achieve sensory awareness)	40 min 3×/week for 12 weeks	Spasticity: MAS	MAS significant results
Bakaniene et al. (2018)	Pre/post cohort study with a conventional therapy control groups Aim: To investigate the effect of the Mollii Suit intervention on gross motor function	SG: 8 spastic CP children (4.6 years old) CG: 8 spastic CP children (4.6 years old)	LL: quadriceps and ankle dorsiflexors	Mollii Suit: 25–175 µs, 20 Hz, NR	60 min 3×/week for 3 weeks	Clinical Assessment: Timed Get and Go, Gross Motor Function Measure Spasticity: MTS pROM: goniometer	Gross Motor Function Measure
Beck et al. (1997)	Case Example Aim: To describe the use of sensory electrical stimulation delivered at night and reinforced by daytime application during therapy	SG: 1 spastic diglegic CP child (9 years old) CG: NA	LL: Quadriceps and M tibialis anterior	TheES: NR, 35–40 Hz, so patient feels tingling	6–7 nights/week for 9 h and 45–60 min 3×/week for 17 months	Clinical Assessment: Ambulation endurance, Gait quality, independence Muscle Strength aROM	Improvement seen in all fields, no significance reported
Dali et al. (2002)	Randomized Double Blind Placebo Controlled Trial Aim: To determine whether a group of stable children with CP would improve their motor skills after 12 months of ThrES	SG: 38 diplegic or hemiplegic CP children (5–18 years old) CG: 19 diplegic or hemiplegic CP children (5–18 years old)	LL: Quadriceps and M tibialis anterior	ThrES: NR, 35 Hz, 1–5 µA (to provide a tickling sensation)	6 h per night, 6 nights/week for 12 months	Clinical Assessment: CT size of muscle Spasticity: Ashworth Scale Muscle Function: Leg ability Test pROM	No significant results reported
Hedin et al. (2022)	Single Arm Study Aim: To evaluate the effect of treatment with the elektrodress for children with CP and functional impairment with spasticity or dystonia	SG: 16 diplegia/ hemiplegia/dystonia CP children CG: NA	Not specified: individually decided based on child's needs	Mollii Suit: 25–175 µs, 20 Hz, NR	60 min, 3–4×/week, for 6 weeks to 1 year (depending on patients)	Spasticity: MAS (1/3/6/12 months), MTS (1/3/6/12 months) pROM: Hip extension/abduction/external rotation, Knee extension/flexion Ankle Dorsal extension (1/3/6/12 months)	MAS (1/6 months) MTS (1 month) pROM (1/3/6/12 months)
Kerr et al. (2006)	Randomized Placebo-Controlled Trial Aim: To investigate the efficacy of NMES and ThrES in strengthening the quadriceps muscles of both legs	SG: 20 CP children CG NMES: 18 CP children CG Placebo: 22 CP children	LL: Quadriceps	ThrES: 300 ms, 35 Hz, sensory threshold (always <10 mA)	8 h, 5 nights/week, for 16 weeks	Clinical Assessment: Gross Motor Function Measure Strength: Quadriceps peak torque Parents Assessment: Lifestyle Assessment Score	Lifestyle Assessment Score showed significant improvement for TES
Liu et al. (2021)	Randomized Controlled Clinical Trial Aim: To investigate the effects of a combination of tPCS (transcranial pulsed current stimulation) and TENS, applied concurrently with multiple stimulating electrodes covering the scalp, spine and lower limbs, would be effective in improving lower limb spasticity in children with SCP categorized on GMFCS levels III–V for long term management of spasticity	SG: 32 spastic CP children (7.63) CG: 31 spastic CP children (9.19)	LL: Cervical/ Thoracal/Lumbal regions of the spine, Adductor longus muscle, Rectus femoris muscle, Gastrocnemius muscle	TENS: 140 µs, 400 Hz, max 10 mA	30 min, 5×/week for 12 weeks	Spasticity: MAS, MTS	MAS, MTS
Logosua et al. (2021)	Quasi Experimental Single Arm Pre/Post Test Design Aim: To evaluate the effectiveness of TENS in the management of calf muscle spasticity	SG: 15 spastic CP children, (1–15 years old) CG: NA	LL: Calf muscles	TENS: 300 µs, 10–20 Hz, 4–20 mA	30 min, 1×	Spasticity: MAS, H reflex amplitude and latency pROM: ankle dorsiflexion	MAS, H reflex amplitude, ankle dorsiflexion
Mäenpää et al. (2004)	Single Arm Clinical Trial Aim: To determine whether add-on ES at the sensory level improves ankle dorsiflexion	SG: 17 CP children (6.4 years old) CG: NA	LL: Tibialis anterior muscle	ES: 300 µs, 10–20 Hz, 4–20 mA	20–60 min, 4–15× in 1 month (individual based on patient)	Clinical Assessment: Standing and hopping on 1 foot (1/2/9 months) Muscle function: Daniels and Worthingham aROM: Ankle dorsiflexion/ inversion/eversion/toe flexion/extension (1/2/9 months) pROM: Ankle dorsiflexion knee flexed/extenden (1/2/9 months)	Standing 1 foot (1/2/9 months), hopping on 1 foot (2/9 months), aROM: Ankle dorsiflexion/ inversion/eversion/toe flexion/extension (1/2/9 months) pROM: ankle dorsiflexion knee flexed (1/2/9 months)
Mäenpää et al. (2004)	Single Arm Clinical Trial Aim: To evaluate the effect of ES on the function of the upper extremities	SG: 12 spastic hemiplegic CP children (5.58 years old) CG: NA	UL: wrist dorsiflexors, 3 children + M triceps brachii, 9 children + M infraspinatus	ES: 300 µs, 20–40 Hz, 2–10 mA	20–40 min, 2–3×/week for 5 weeks	Spasticity: Zancolli classification Muscle function: Daniels and Worthingham Bimanual function: King's hypertonicity scale Goal Attainment Scale	Zancolli classification, Daniels and Worthingham supination ability: Beginning with arm flexed, with arm extended for children over 4 years
Pape et al. (1997)	Single Subject Design Clinical Trial Aim: To determine whether the beneficial effects of ES could reduce spasticity for the long term	SG: 6 CP children (4.24 years old) CG: NA	LL: Tibialis anterior muscle and quadriceps	ES: 300 µs, 35–45 Hz, <10 mA	9 h a night, 6 months on, then 6 months off, then 6 months on again	Clinical Assessment: Peabody Developmental Motor Scales	Peabody Developmental Motor Scales: first 6 months: total gross motor, locomotor, and receipt/ propulsion skills, second 6 months: total gross motor, balance, locomotor, and receipt/propulsion
Pool et al. (2020)	Randomized Controlled Trial Aim: To determine if robotic assisted gait training (RAGT) using surface muscle ES and locomotor training enhances mobility outcomes when compared to locomotor training alone	SG: 20 CP children (8.33 years old) CG: 20 CP children (8.1 years old)	LL: Quadriceps and hamstrings	Robotic Assisance + ES: starting with 50 µs, 50 Hz, NR	20 min, 3×/week, for 6 weeks	Clinical Assessment: 10 m Walking Test, Children's Functional Independence Measure: mobility and self care domain, Gross Motor Function Measure, Canadian Occupational Performance Measure	No significant results reported
Satheeskumar et al. (2018)	Randomised, Placebo-Controlled and Multi-Centered Study Design Aim: To find the effectiveness of TENS combination with sensorimotor task-oriented training to improve hand function	SG: 30 unilateral CP children (6.02 years old) CG: 30 unilateral CP children (6.5 years old)	UL: Forearm flexor and extensor group muscles	TENS: 200 µs, 100 Hz, 2–3× sensory threshold	60 min, 3×/week for 8 weeks	Clinical Assessment: Tactile Localisation and Resgistration, 2 Point Discrimination, Stereognosis Unimanual Hand Performance: Quest Score, 9 Hole Peg Test Parents Assessment: ABILHAND Kid's	Tactile Localisation and Registration, Quest Score, 9 Hole Peg Test
Sommerfelt et al. (2001)	Crossover Study Aim: To evaluate the effect of TheES applied to antagonists of spastic leg muscles on gross motor function	SG: 12 spastic diplegic CP children (5–12 years old) CG: Same as SG (crossover design)	LL: Quadriceps and tibialis anterior muscle	TheES: 300 µs, 40 Hz, <10 mA	mininum 5 h/night, 6×/week for 6 months	Clinical Assessment: 6-metre walk, 1-minute weight shifts, 6-minute walk, Standing balance, Walking line, Sitting balance Deep tendon reflexes, Peabody Developmental Motor Scales: Balance and Locomotor Strength: Quadriceps, Tibialis anterior muscle, Gastrocnemius (all both sides) pROM: ankle dorsiflexion	No significant results reported
Steinbok et al. (1997)	Randomized Controlled Single Blind Trial Aim: to determine the effectiveness of TheES in improving the function of children with spastic CP, who had undergone selective posterior lumbosacral rhizotomy more than a year previously.	SG: 20 spastic CP children (7.2 years old) CG: 21 spastic CP children (7.3 years old)	LL: abdominal, Gluteus medius and maximus muscles, Quadriceps, Tibialis anterior muscle	TheES: 300 ms, 35 Hz, <10 mA	8–12 h/night, minimum 6 nights/week for 12 months	Clinical Assessment: Seated posture control Spasticity: MAS Strength: Hip abduction, Knee extension, Ankle dorsiflexion pROM: Hip abduction, Knee extension, Ankle dorsiflexion	Seated Posture Control
Sultan et al. (2005)	Pre Post Study Aim: to evaluate the effect of botulinum toxin A (BTX-A), TENS and kinesthetic gait training in managing lower limb spasticity	SG: 12 diplegic CP children (4.67 years old) CG: -Botox: 9 diplegic CP children (4.50) -Treadmill: 14 diplegic CP children (4.27) -Gait Training: 12 diplegic CP children (4.53)	LL: Fibularis muscles, extensor muscles of the lower leg	TENS: 125 µs, 99 Hz, without causing muscle contraction	30 min; 3×/week for 6 months	Clinical Assessment: Activity of daily living by mobility section of the barthel. Posture by equilibrium scoring during standing on the force platform, Gait parameters: velocity, cadence and stride length Spasticity: MAS	Activity of daily living by mobility section of the barthel, velocity of gait, MAS

CP, cerebral palsy; SG, study group; CG, control group; UL, upper limbs; LL, lower limbs; NR, not reported; NA, not applicable; MAS, modified Ashworth scale; MTS, modified tardieu scale; pROM, passive range of motion; aROM, active range of motion; TheES, therapeutic electrical stimulation; ThrES, therapeutic electrical stimulation; NMES, neuromuscular electrical stimulation.

### 2.7 Risk of bias assessment

The Risk of Bias (ROB) assessment was completed by two independent reviewers (CdA, SG). For RCTs the Revised Cochrane risk-of-bias tool for randomized trials (RoB 2) was used, evaluating five domains: randomization process, deviations from intended interventions, missing outcome data, measurement of outcomes, and selection of reported results (22). The overall risk of bias was categorized as low risk, some concern, or high risk, with results shown in Table 2. For retrospective studies and those without control groups, the National Institute of Health (NIH) Quality Assessment Tool for Before-After (Pre-Post) Studies With No Control Group was applied. This tool assesses twelve domains including the study question, eligibility criteria, representation of the study population, enrollment of eligible participants, sample size, clarity of intervention and outcome measures, blinding of assessors, follow-up rates, statistical analysis, multiple outcome measures, and interventions at both group and individual levels. The overall risk of bias was rated as good, fair, or poor (23). These results are shown in Table 3. One of the included articles was a case study (14) where no ROB was conducted. Discrepancies in assessment were discussed and resolved (CdA and SG). None of the studies were excluded based on the ROB assessment.

**Table 2:** Risk of bias of the included studies based on the revised Cochrane risk-of-bias tool for randomized trials.

Revised Cochrane risk-of-bias tool for randomized trials	Alabdulwahab et al. 2010 (24)	Alhusaini et al. 2019 (11)	Azzam et al. 2012 (13)	Dali et al. 2002 (15)	Kerr et al. 2006 (27)	Liu et al. 2021 (28)	Pool et al. 2020 (29)	Satheeskumar et al. 2018 (26)	Steinbok et al. 1997 (30)
Domain 1: Risk of bias arising from the randomization process									
1.1 Was the allocation sequence random?	NI	Y	Y	Y	Y	Y	Y	Y	Y
1.2 Was the allocation sequence concealed until participants were enrolled and assigned to interventions?	NI	NI	NI	Y	Y	Y	Y	Y	Y
1.3 Did baseline differences between intervention groups suggest a problem with the randomization process?	Y	N	N	N	PY	PY	N	N	N
Domain 1 judgement	Some concerns	Low risk risk	Low risk	Low risk	Low risk	Low risk	Low risk	Low risk	Low risk
Domain 2: Risk of bias due to deviations from the intended interventions (effect of assignment to intervention)									
2.1. Were participants aware of their assigned intervention during the trial?	Y	Y	Y	N	N	Y	Y	N	Y
2.2. Were carers and people delivering the interventions aware of participants’ assigned intervention during the trial?	Y	Y	Y	N	N	Y	Y	N	Y
2.3. If Y/PY/NI to 2.1 or 2.2: Were there deviations from the intended intervention that arose because of the trial context?	N	N	N	NA	N	PY	NI	NA	N
2.4 If Y/PY to 2.3: Were these deviations likely to have affected the outcome?	NA	NA	NA	NA	NA	PY	NA	NA	NA
2.5. If Y/PY/NI to 2.4: Were these deviations from intended intervention balanced between groups?	NA	NA	NA	NA	NA	N	NA	NA	NA
2.6 Was an appropriate analysis used to estimate the effect of assignment to intervention?	Y	Y	PY	Y	Y	Y	Y	Y	Y
2.7 If N/PN/NI to 2.6: Was there potential for a substantial impact (on the result) of the failure to analyse participants in the group to which they were randomized?	NA	NA	NA	N	NA	NA	NA	NA	NA
Domain 2 judgement	High risk	Some concerns	Some concerns	Low risk	Low risk	Some concerns	Some concerns	Low risk	Some concerns
Domain 2: Risk of bias due to deviations from the intended interventions (effect of adhering to intervention)									
2.1. Were participants aware of their assigned intervention during the trial?	Y	Y	Y	N	N	Y	Y	N	Y
2.2. Were carers and people delivering the interventions aware of participants’ assigned intervention during the trial?	Y	Y	Y	N	N	Y	Y	N	Y
2.3. [If applicable:] If Y/PY/NI to 2.1 or 2.2: Were important non-protocol interventions balanced across intervention groups?	NI	NI	NI	NA	NA	NI	Y	NA	Y
2.4. [If applicable:] Were there failures in implementing the intervention that could have affected the outcome?	N	N	N	N	N	PY	N	N	N
2.5. [If applicable:] Was there non-adherence to the assigned intervention regimen that could have affected participants’ outcomes?	NI	N	N	N	N	PY	N	N	Y
2.6. If N/PN/NI to 2.3, or Y/PY/NI to 2.4 or 2.5: Was an appropriate analysis used to estimate the effect of adhering to the intervention?	NI	Y	Y	NA	NA	Y	NA	NA	NA
Domain 2 judgement	High risk	Some concerns	Some concerns	Low risk	Low risk	Some concerns	Some concerns	Low risk	Some concerns
Domain 3: Missing outcome data									
3.1 Were data for this outcome available for all, or nearly all, participants randomized?	PN	Y	Y	Y	Y	Y	Y	Y	Y
3.2 If N/PN/NI to 3.1: Is there evidence that the result was not biased by missing outcome data?	PY	NA	NA	NA	NA	NA	NA	NA	NA
3.3 If N/PN to 3.2: Could missingness in the outcome depend on its true value?	PY	NA	NA	NA	NA	NA	NA	NA	NA
3.4 If Y/PY/NI to 3.3: Is it likely that missingness in the outcome depended on its true value?	N	NA	NA	NA	NA	NA	NA	NA	NA
Domain 3 judgement	Some concerns	Low risk	Low risk	Low risk	Low risk	Low risk	Low risk	Low risk	Low risk
Domain 4: Risk of Bias in measurement of the outcome									
4.1 Was the method of measuring the outcome inappropriate?	N	N	N	N	N	N	N	N	N
4.2 Could measurement or ascertainment of the outcome have differed between intervention groups?	N	N	NI	N	N	N	N	N	N
4.3 If N/PN/NI to 4.1 and 4.2: Were outcome assessors aware of the intervention received by study participants?	NI	NI	NI	N	N	N	N	N	N
4.4 If Y/PY/NI to 4.3: Could assessment of the outcome have been influenced by knowledge of intervention received?	Y	Y	Y	NA	NA	NA	NA	NA	NA
4.5 If Y/PY/NI to 4.4: Is it likely that assessment of the outcome was influenced by knowledge of intervention received?	N	N	N	NA	NA	NA	NA	NA	NA
Domain 4 judgement	Some concerns	Some concerns	Some concerns	Low risk	Low risk	Low risk	Low risk	Low risk	Low risk
Domain 5: Risk of bias in selection of the reported result									
5.1 Were the data that produced this result analysed in accordance with a pre-specified analysis plan that was finalized before unblinded outcome data were available for analysis?	NI	NI	NI	NI	NI	NI	NI	NI	NI
5.2 Is the numerical result being assessed likely to have been selected, on the basis of the results, from multiple eligible outcome measurements (e.g., scales, definitions, time points) within the outcome domain?	Y	Y	N	Y	Y	Y	Y	N	PY
5.3 Is the numerical result being assessed likely to have been selected, on the basis of the results, from multiple eligible analyses of the data?	N	N	N	N	N	N	NI	N	NI
Domain 5 judgement	Some concerns	Low risk	Low risk	Low risk	Low risk	Low risk	Low risk	Low risk	Some concerns
Overall Rob Judgement	High risk	Some concerns	Some concerns	Low risk	Low risk	Some concerns	Some concerns	Low risk	Some concerns

Y, yes; PY, partially yes; PN, partially no; N, no; NA, not applicable, NI, no information.

**Table 3:** Risk of bias of the included studies based on the NIH quality assessment tool for before-after (Pre-post) studies With No control group.

NIH quality assessment tool for before-after (pre-post) studies with no control group	Bakaniene et al. 2018 (33)	Hedin et al. 2022 (36)	Logosua et al. 2021 (32)	Mäenpää et al. 2004 (31)	Mäenpää et al. 2004 (25)	Pape et al. 1997 (34)	Sommerfelt et al. 2001 (16)	Sultan et al. 2005 (35)
1. Was the study question or objective clearly stated?	Y	Y	Y	Y	Y	Y	Y	Y
2. Were eligibility/selection criteria for the study population prespecified and clearly described?	Y	Y	Y	Y	Y	Y	Y	Y
3. Were the participants in the study representative of those who would be eligible for the test/service/intervention in the general or clinical population of interest?	Y	Y	Y	Y	Y	Y	Y	Y
4. Were all eligible participants that met the prespecified entry criteria enrolled?	N	NR	NR	Y	Y	NR	N	NR
5. Was the sample size sufficiently large to provide confidence in the findings?	N	N	N	N	N	N	N	N
6. Was the test/service/intervention clearly described and delivered consistently across the study population?	Y	Y	Y	Y	Y	Y	Y	Y
7. Were the outcome measures prespecified, clearly defined, valid, reliable, and assessed consistently across all study participants?	Y	Y	Y	Y	Y	Y	Y	Y
8. Were the people assessing the outcomes blinded to the participants’ exposures/interventions?	N	N	NR	NR	Y	Y	Y	NR
9. Was the loss to follow-up after baseline 20% or less? Were those lost to follow-up accounted for in the analysis?	Y	N, N	NR	Y	Y	Y	Y	Y
10. Did the statistical methods examine changes in outcome measures from before to after the intervention? Were statistical tests done that provided *p*-values for the pre-to-post changes?	Y, Y	N, Y	Y, Y	Y, Y	Y, Y	N, Y	Y, N	N, Y
11. Were outcome measures of interest taken multiple times before the intervention and multiple times after the intervention (i.e., did they use an interrupted time-series design)?	N	N	N	Y	Y	N	N	N
12. If the intervention was conducted at a group level (e.g., a whole hospital, a community, etc.) did the statistical analysis take into account the use of individual-level data to determine effects at the group level?	NA	NA	NA	NA	NA	NA	NA	NA

Y, yes; N, no; NR, not reported; NA, not applicable.

## 3 Results

### 3.1 Study selection

The database search identified 504 results. After completing the citation searching and removing duplicates, 338 articles remained to undergo title and abstract screening, from which 270 were excluded, leaving 68 titles for the full-text screening. Upon completing this process, 18 studies met the inclusion criteria and were therefore included in this review. Most articles that were excluded after full-text screening were due to the applied ES triggering muscle contractions. The detailed screening process is shown in the PRISMA flowchart in Figure 1.

### 3.2 Study characteristics

Of the 18 studies published between 1997 and 2022 that were included, nine were RCTs. When evaluating all the studies evaluated by this scoping review, a total of 564 children were included with an age range of 1–18 years. Of these, 549 were children diagnosed with CP while the remaining 15 were healthy children used as controls by AlAbdulwahab et al. (24). The CP diagnoses included hemiplegic, diplegic and quadraplegic spastic forms as well as dystonic and ataxic forms. A total of 316 children underwent one of the ES forms described above. Detailed information on each study is provided in the online supporting information, with a summary of this information found in Table 1.

### 3.3 Risk of bias assessment

According to Cochrane's RoB 2 for clinical trials, the major sources of bias arose due to deviations from the intended interventions, both in terms of the effect of adhering to the intervention as well as due to the effects of assignment to intervention. The NIH ROB tool revealed the following as the main sources of bias: lack of large enough sample size to provide confidence, lack of blinding outcome assessors and failure to measure outcome measures multiple times before and after the interventions.

### 3.4 Regional applications of SAES

In the reviewed articles, three distinct areas of implementation in which SAES were used were identified: SAES applied to the lower extremities, SAES applied to the upper extremities and SAES involving the entire body using a Mollii Suit. The primary indications for SAES in these different body regions were either the reduction of spasticity or the improvement of specific functional capacities within the respective body areas, such as strength, function, or mobility. In the following sections, the specific indications for each body region are described in detail, structured according to the region of the body in which it has been applied.

### 3.5 Upper limbs

#### 3.5.1 Study types

A total of four articles were included in this review that looked into the effects of SAES on the upper extremities (11, 13, 25, 26). These included three RCT (11, 13, 26) and one single arm clinical trial (25).

#### 3.5.2 Stimulation forms and parameters

Of the four articles studying the effects on the upper limbs, two used TENS (11, 26), while Azzam et al. (13) used a Mesh Glove and Mäenpäa et al. (25) described using ES, without any further specifications of which form. The pulse duration was very similar for all four patient groups, including 200 µs (26), 250 µs (11) and 300 µs (13, 25). Intensity varied from between 2 and 10 mA (13, 25) and 50 mA (11), with Satheeskumar et al. (26) setting the intensity individually at two to three times the child's sensory threshold all while ensuring the stimulation didn't exceed a tingling sensation reported by the child. The frequency of the stimulation was reported to be either between 20 and 40 Hz (13, 25) or 100 Hz (11, 26).

Treatment lasted between five weeks (25) and twelve weeks (13), with Alhusaini et al. (11) and Satheeskumar et al. (26) conducting stimulation for eight weeks. The most common frequency of ES was for 30 min two to three times a week (11, 13, 25), with Satheeskumar et al. (26) describing sessions of 60 min three times a week.

#### 3.5.3 Outcome measures

The four articles from above described a range of different outcome measurements. Alhusaini et al. (11) aimed to see a reduction in spasticity leading to an increase in hand function, which was measured using hand grip strength, the Jebsen Taylor Hand Function Test (measuring unimanual performance) as well as the ABILHAND-Kids to measure the child's ability to manage daily activities. The first of these two evaluation methods showed significant improvements, with the ABILHAND Questionnaire reporting non significant results. Azzam et al. (13) measured the effect of Mesh Glove stimulation on spasticity using the Modified Ashworth Scale (MAS) and showed significant improvement from baseline to post treatment. Mäenpää et al. (25) sought to improve muscle function and measured spasticity using Zancolli's classification, muscle function using the Daniels and Worthingham Muscle Testing, bimanual hand function using the King's Hypertonicity Scale and measured the childrens' progress using the Goal Attainment Scale. Of these, significant improvements were seen in flexed arm testing according to Daniel and Worthingham Muscle Function, while the extended arm testing showed no statistical significance. Zancolli's classification showed significant improvements immediately after the electrical stimulation period as well as three months after the cessation of electrical stimulation. King's Hypertonicity Scale showed a significant change in hand function in each age group, with some individuals switching to using the affected hand as their dominant hand in tasks such as the screw-top test. Satheeskumar et al. (26) endeavored to evaluate the effect of TENS on improving hand function utilizing the following tests to measure the results: Tactile Registration and Localisation as well as Two Point Discrimination and Stereognosis clinical tests, with the first two showing significant results and the last two being non-significant. To assess unimanual hand function the Quest Score and Nine Hole Peg Test were used, both showing significant results. The ABILHAND-Kids Questionnaire was also used and showed non-significant results.

### 3.6 Lower limbs

#### 3.6.1 Study types

A total of thirteen studies collected for this review examined the effects of ES on lower limbs. Of these, six were RCT (15, 24, 27–30). The remaining seven included: two single arm clinical trial (31, 32), one pre/post cohort study (33), one single subject clinical trial (34), one crossover study (16) and one unspecified study type (35).

#### 3.6.2 Stimulation forms and parameters

The stimulation modalities used on the lower extremities were most often TENS (24, 28, 32, 35) with a total of four, followed by three studies using TheES (14, 16, 30) another three articles using ES (29, 31, 34), two other articles using ThrES (15, 27) and finally one using the Mollii Suit (33). Pulse duration ranged greatly from a minimum of 25 µs (33) ranging all the way up to 300 ms (30). Intensity was defined so that the patient could feel a tingling or to not create a contraction in six studies (14, 15, 24, 27, 32, 35), four specified that it remained under 10 mA (16, 28, 30, 34), Mäenpäa et al. (31) reported a range from 4 to 20 mA, with the two remaining studies not specifyng how intensity was measured (29, 33). Frequencies ranged from 10 Hz (31) to 400 Hz (28).

Treatment span varied greatly with some lasting only a single session (24, 32) with others administered repeatedly over the course of seventeen months (14). The duration of the individual stimulation periods differed from 15 min (24) to nine hours (34).

#### 3.6.3 Outcome measures

One of the most common outcome measures was the effect on spasticity in a total of seven articles (15, 24, 28, 30, 32, 33, 35). The Modified Ashworth Scale (MAS) was the most frequently used tool to assess changes in spasticity, used in five of these articles (24, 28, 30, 32, 35) and showing significant results in four of these (24, 28, 32, 35). The Modified Tardiu Scale (MTS) was used in two articles (28, 33) with Liu et al. (28) showing significant results in terms of reducing spasticity when testing the quality of muscle reaction and passive (pROM) over the ankle and knee joints as well as of the adductor muscles. Bakaniene et al. (33) showed no meaningful change in the spasticity of the ankle plantar flexors and hamstrings in the experimental and control groups. Dali et al. (15) used the Ashworth Scale to measure spasticity, showing no significant results while Logosua et al. (32) was able to produce significant results regarding spasticity when measuring the H reflex amplitude, although not in the H reflex latency, indicating some reduction in spasticity.

Four articles measured muscle strength as an outcome measure, with none of the measurements resulting in significant improvements (14, 16, 27, 30).

Active range of motion (aROM) was used as an outcome variable by Beck et al. (14) without yielding significant improvements regarding ankle dorsiflexion, while measurements completed by Mäenpäa et al. (31) were able to show significant results concerning ankle dorsiflexion, inversion, eversion as well as toe flexion and extension.

Passive range of motion (pROM) was also used as an outcome measure for six articles (15, 16, 30–33), with two of these showing significant improvements in ankle dorsiflexion (31, 32). The Goal Attainment Scale was applied in two studies, with neither of them showing significant improvements (29, 31).

### 3.7 Whole body stimulation

#### 3.7.1 Study types

One single arm study included did not specify if the ES was applied to the upper or lower extremities (36).

#### 3.7.2 Stimulation parameters

In this paper the Mollii Suit was used with a pulse duration of 25–175 µs, a frequency of 20 Hz and an unspecified intensity. Treatment involved sessions lasting 60 min, administered three to four times a week ranging from six weeks to a year, depending on the patient in question (36).

#### 3.7.3 Study types outcome measures

Spasticity was measured as an outcome using the MAS and MTS, the MAS showed significant results one and six months after treatment regardig hip adductors, knee flexors and plantar flexors, with insignificant results at three months. The MTS showed significant results one month after treatment for hip flexors and hip adductors as well as plantar flexors. pROM also showing significant results one, three, six and twelve months after treatment end in hip external rotation, knee extension, hip abduction and hamstrings (36).

### 3.8 Side effects of all SAES treatment

The most commonly reported side effect of the ES treatments were skin redness or rashes in the location of the electrode placements, occurring in a total of seven children (15, 27, 28, 34). A total of three children dropped out of the treatment program due to this skin irritation (15, 27). Hedin et al. (36) reported several children that struggled putting the Mollii Suit on as it was described as being very tight.

Certain studies asked for the appraisal of the parents outside of a standardized questionnaire, with the majority of families reporting the ES therapies easy to use and their expectations being met (15, 25, 27, 31, 34, 36). Some parents even reported seeing an increase in awareness of the affected limb after these ES therapies (25), a decrease in day time spasticity (34) as well as the development of new skills such as independent cycling (31).

## 4 Discussion

This review aimed to map the available literature on the application of SAES in children with CP. The screening process left a total of 18 studies that fulfilled the predefined eligibility criteria. These studies showcase various modalities of SAES, including TENS, TES, TheES, ThrES and innovative approaches such as the Mollii suit. These techniques present promising methods for improving motor functions and reducing spasticity, focusing on three main indications: upper extremities, lower extremities, and whole-body treatments.

The application of SAES for treating upper extremities is particularly promising, as this indication is often associated with functional limitations in daily life. Studies show that ES of the upper extremities has numerous beneficial effects on participants. Researchers have documented that children receiving SAES exhibit improved hand function and increased hand strength, as well as experiencing a reduction in spasticity (11, 13, 25, 26). These improvements can enhance not only the independence of children but also their self-esteem, social integration and ability to master everyday tasks.

The treatment of lower extremities with SAES has proven an interesting component to target in order to promote walking ability and balance in children with CP. Most studies that tested the effect of SAES on spasticity in lower limbs indicated that the treatment lead to a decrease therein (24, 28, 32, 35), which is particularly significant considering the impact spasticity has on the independence and mobility of these children. Strength of the lower extremities seemed to not be affected by the use of SAES (14, 16, 27, 30), contrasting results found for the upper extremities, warranting further investigation. Range of motion (ROM) showed some improvements in both passive and active capacities (14, 31, 32), which could play an important contributing role to mobility. The decreased spasticity coupled with increased ROM and strength following SAES provide the potential for substantial positive effects on a child's activities and participation in everyday tasks.

The Mollii suit treatment represents an innovative approach that allows for whole-body stimulation. This suit was designed to provide stimulation across large areas, which can result in improvements in overall motor function, muscle tone, and body awareness. Of the two studies included looking at the effects of the Mollii suit, one was able to demonstrate a reduction in spasticity and an increase in ROM (36), while the other failed to show this reduction of spasticity but was able to show improvements in mobility (33). These effects show the potential of this treatment form, however due to the low number of studies and participants included in them, more investigation is warrented to come to conclusions about the role of the Mollii suit in the treatment of children with CP.

In general, the stimulation approaches presented in this article showed a wide range of applications in terms of indications and outcomes, but also in terms of stimulation parameters and time spent administering the therapy. While this variability does present challenges regarding comparisons, it may also provide benefits. Children with CP are known to be a very heterogeneous population in terms of disease manifestation and characteristics, and the ability to adapt treatment methods may allow catering to this wide range of abilities and symptoms and therefore offer an individualized and holistic therapy. It is however important to note that in order to draw more far-reaching conclusions, more investigation in the form of high standard study designs such as RCTs needs to be performed into these different parameters to allow for the determination of the best approaches for the respective populations.

The high tolerability of SAES is another promising aspect, as only a few side effects, such as temporary skin irritations, have been documented, supporting its application in clinical practice.

Despite the positive results reported by many studies on SAES in children with CP, there remains a need for critical consideration of methodology and potential biases in research. The use of Cochrane risk assessment tools to analyse the included studies has revealed that some studies exhibit a risk of systematic bias. Common issues include inadequate randomization, lack of blinding, and incomplete reporting of results. When using the NIH ROB tool, bias was identified to arise from insufficient sample sizes to provide confidence in findings, which was not the case in any of the included studies. In addition, only three papers of those analysed had the assessors blinded to the participants interventions, increasing the risk of observer bias in the remaining studies. This promotes a non-representative sample, limiting the ability to generalize findings to the larger population of children with CP while creating a tendency to overestimate improvements in the treatment group while underestimating progress in the other treatment groups. In contrast, the eligibility criteria were clearly defined in all the studies, with outcome measures prespecified and clearly and consistently defined, reducing selection and reporting bias as well as aspects of measurement bias, and raising the reliability of the reported results.

Additionally, we observed that the statistical methods used in some articles raise concerns. For example, confidence intervals were only included in four of the eighteen studies (16, 27, 29, 30). This leads to a reduction of the precision of results presented and lead to an overreliance on p values, without further consideration of the consistency of the results. A power analysis for the study size was also often overlooked and only performed in six of the papers (11, 26–30). This uncertainty regarding the sample sizes and if these were large enough to detect meaningful effects produces results that are hard to interpret in the larger clinical setting. These methodological limitations are important to acknowledge to encourage more robust and transparent research in the future. Of the two papers that included a confidence interval, effect size and performed a power analysis of the sample size (27, 29) and therefore the most robust statistical analysis, Kerr et al. (27) showed significant improvements in those children who had undergone ThrES. This suggests that the results provided show improvement even when undergoing proper statistical rigor, which would need to be continued in future studies.

The present article has a variety of strengths and limitations. As far as we are aware, it is the first paper that summarizes the effects of different forms of SAES in children with CP. The study selection was conducted over a span of 25 years, allowing the reviewers to observe these stimulation methods across a wide variety of settings.

One major challenge lies in the variety of terminology used to refer to SAES, making it difficult to comprehensively include all relevant research in the search. Although we incorporated a wide range of terms in our search strategy, it remains possible that we overlooked articles due to the use of different terminology for SAES. Furthermore, the absence of specific definitions or parameters for different types of SAES complicates the grouping of these methods for evaluation.

In addition to varying terminology, a large span of stimulation parameters were used as well, with pulse duration ranging from 50 µs (29) to 300 ms (30), and frequencies from 10 Hz (31) to 400 Hz (28). These variations makes comparing the effects of the different stimulations and their clinical relevance difficult. Initial comparisons suggest that spasticity is most significantly improved according to the MAS when using a stimulation range from 125 µs–300 µs. (13, 24, 28, 32, 35, 36) However, with the fact that these stimulation forms are performed in diverse environments and range from one session (24) to six hours a night, six nights a week for a year (15), coupled with differing frequencies and amplitudes makes a direct comparision nearly impossible.

The articles included in this scoping review displayed a general heterogeneity in respect to the broad range of parameters, particularly regarding pulse duration and frequency. This variance limits the ability to compare outcomes across studies effectively and necessitates further investigation into the specific parameters that may influence treatment efficacy. Participants included varied widely in terms of age, severity of disability and comorbidities, making it challenging to draw general conclusions and assess the transferability of results to broader patient groups. Due to this great heterogentiy as well as the diverse ways in which the disability grade of the children was reported, a further stratification of results according to the children's physical abilites was not performed, a further limitation and one that would need to be studied in more depth in later research. Children with CP represent an extremely diverse group of patients. Variations in disease presentation can significantly affect responses to treatments in that children with milder forms of CP may respond differently to SAES compared to those with more severe impairments. These differences necessitate a nuanced consideration when evaluating the effectiveness of SAES and suggest that individualized therapy approaches are essential to address the varying needs of patients.

Additionally, during the screening process, several studies failed to clarify whether the ES used induced muscle contractions. Some attempts to contact the authors for clarification were unsuccessful, leading to the exclusion of potentially valuable sources. Furthermore, several included studies did not provide detailed explanations for patient dropouts, which could introduce bias in the reported outcomes. Some studies also employed personalized training programs tailored to each individual child, with varying durations of ES treatment. This lack of standardization complicates the examination of results and diminishes the reliability of the conclusions drawn.

Moreover, we limited our review to published articles from peer-reviewed journals, focusing solely on those available in languages with which we were familiar. As a result, we did not include grey literature or studies published in other languages, which may have led to the omission of relevant articles and insights.

## 5 Conclusion

In conclusion, this review revealed that the research available on SAES in children with CP is highly diverse and encompasses various indications, parameters and effects. While the current evidence suggests that SAES shows promise for improving motor skills in children with CP, the great diversity in study designs and outcomes limits direct comparisons and prevents definitive conclusions. Further high-quality studies are necessary to clarify optimal applications, underlying mechanisms, and the long-term effects of this therapeutic approach. In order to make this a reality studies would need to performed using different stimulation parameters in comparison to determine the frequency and pulse duration with the most effect on predetermined outcome measures, for example spasticity. In a second step the environment in which the stimulation occurs could be compared, with one group undergoing the treatment for short stretches throughout the day while others experienced it overnight for several hours on end. This could be built upon in that children with different disability levels were compared in order to determine if for example the ability to ambulate has an effect on progress made with stimulation therapies. This review highlights the fact that SAES remains an emerging field, emphasizing the clear need for further rigorous research with standardized methodologies to allow it to gain stronger evidence and establish its role as a therapeutic intervention.
