# Supplementary material for: Sensory-level electrical stimulation in children with cerebral palsy: a scoping review of current applications and outcomes
Source: Front Pediatr. 2025 Nov 24;13:1644547. doi: 10.3389/fped.2025.1644547 (PMC12682778; doi:10.3389/fped.2025.1644547)
Supplement: Supplementary file 1 [file Table1.docx]

Table A1. Search Terms used

Ti=title, ab=abstract, kw=keyword, exp=exploded, de=not exploded

| Database | Population | Exposure |
| --- | --- | --- |
| Medline by Ovid | Cerebral Palsy [Mesh] OR ("cerebral pals*" or "spastic quadriplegi*" or "spastic tetraplegi*" or "CP" or "mixed cerebral pals*" or "quadriplegic cerebral pals*" or "spastic cerebral pals*" or "spastic diplegi*" or "athetoid cerebral pals*").ti,ab,kw.  AND  Child [Mesh] OR Adolescent [Mesh] OR Pediatrics [Mesh] OR Infant [Mesh] OR Newborn [Mesh] OR ("pediatric*" or "child*" or "adolescent*" or "teen*" or "toddler*" or "youth*" or "juvenil*" or "infant*").ti,ab,kw. | Electrical Stimulation [Mesh] OR Transcutaneous Electric Nerve Stimulation [Mesh] OR ("whole-hand afferent electrical stim*" or "mollii suit" or "whole hand electrical stim*" or "mesh-glove afferent electrical stim*" or "afferent electric* stim*" or "somatosensory electrical stim*" or "sensory afferent electrical stim*" or "electrostim* at sensory level*" or "mesh glove sensory stim*" or "therapeutic electric* stim*" or "transcutaneous electric* nerve stim*" or "TENS" or "TES").ti,kw,ab. |
| Embase by Elsevier | 'cerebral palsy'/exp OR 'cerebral pals*':ti,ab,kw OR 'spastic quadriplegi*':ti,ab,kw OR 'spastic tetraplegi*':ti,ab,kw OR 'cp':ti,ab,kw OR 'mixed cerebral pals*':ti,ab,kw OR 'quadriplegic cerebral pals*':ti,ab,kw OR 'spastic cerebral pals*':ti,ab,kw OR 'spastic diplegi*':ti,ab,kw OR 'athetoid cerebral pals*':ti,ab,kw  AND  'child'/de OR 'adolescent'/de OR 'pediatric'/de OR 'toddler'/de OR 'juvenile'/de OR 'pediatric*':ti,ab,kw OR 'child*':ti,ab,kw OR 'adolescent*':ti,ab,kw OR 'teen*':ti,ab,kw OR 'toddler*':ti,ab,kw OR 'youth*':ti,ab,kw OR 'juvenil*':ti,ab,kw | 'electrostimulation'/exp OR 'transcutaneous electrical nerve stimulation'/exp OR  'whole-hand afferent electrical stim*':ti,kw,ab OR 'mollii suit':ti,kw,ab OR 'whole hand electrical stim*':ti,kw,ab OR 'mesh-glove afferent electrical stim*':ti,kw,ab OR 'afferent electric* stim*':ti,kw,ab OR 'somatosensory electric* stim*':ti,kw,ab OR 'sensory afferent electrical stim*':ti,kw,ab OR 'electrostim* at sensory level*':ti,kw,ab OR 'mesh glove sensory stim*':ti,kw,ab OR 'therapeutic electric* stim*':ti,kw,ab OR 'tes':ti,kw,ab OR 'tens':ti,kw,ab |
| PEDro | Dropdown Menus:  -Topic: Cerebral Palsy  -Subdiscipline- Pediatrics | Abstract & Title: Electrical Stimulation |
